# Atypical Bilateral Proximal Femoral Focal Deficiency: A Case Report and Review of Literature

**DOI:** 10.1155/crpe/7521784

**Published:** 2026-06-08

**Authors:** Ahmad Hammad, Haya El Merkabaoui, Hicham Moukaddam, Mohammad Kassem, Claudia El Haddad, Rachid Haidar

**Affiliations:** ^1^ Department of Orthopedics Surgery, American University of Beirut Medical Center, Beirut, Beirut Governate, Lebanon, aubmc.org.lb; ^2^ Department of Diagnostic Radiology, American University of Beirut Medical Center, Beirut, Beirut Governate, Lebanon, aubmc.org.lb

**Keywords:** limb length discrepancy, limb lengthening, orthopedic surgery, prosthetics, proximal femoral focal deficiency

## Abstract

**Introduction:**

Proximal femoral focal deficiency (PFFD) is a rare congenital skeletal abnormality with an incidence of 1 per 100,000 births. It is typically a unilateral abnormality but can present bilaterally in 15% of PFFD cases. It is characterized by the absence of the proximal femur and shortening of the affected limb henceforth. It is associated with other skeletal anomalies, and its radiographic appearance continues to progress with age. Treatment options vary widely from conservative approaches to surgical correction and stabilization. This report presents a case description and a review of diagnostic and management approaches.

**Case presentation:**

A 2‐year‐old girl presented with abnormal crawling, standing, and walking postures. On physical examination, the patient had unusual short bulky thighs with an apparent limb length discrepancy of 2.5 cm and the hips positioned in flexion, abduction, and external rotation, with bilateral knee flexion contractures. Radiographs showed focal deficiency of the proximal femurs bilaterally and shortening. MRI revealed bilateral severely dysplastic acetabula with dislocation of bilateral femurs and formation of pseudoacetabula. PFFD on the right is consistent with Aitken C (absent femoral head) and on the left with Aitken B (femoral head present but without an osseous connection between the head and shaft).

**Conclusion:**

Bilateral PFFD is a rare entity that is associated with a myriad of difficulties. It is crucial to acknowledge the importance of imaging‐based diagnosis and individualized management tailored to imaging findings, possibly even within the same patient with bilateral asymmetric PFFD. A multidisciplinary approach is needed to discuss the treatment options available, aiming to restore a functional limb of adequate length and maintain quality of life.

## 1. Introduction

Proximal femoral focal deficiency (PFFD) is a rare congenital deficiency of the proximal femur that can extend from the femoral head to the midshaft, with an incidence of 1 in every 100,000 newborns [1, 2]. It is typically unilateral but involves bilateral femurs in 15% of affected children. PFFD belongs to a broad spectrum of femoral deformities referred to as congenital femoral deficiency (CFD) as defined by Paley and Nossov. The CFD spectrum encompasses a range of femoral deformities characterized by varying degrees of shortening, malrotation, and deficiency of the proximal femur and acetabulum [3] and hence the association between PFFD and accompanying lower limb deformities [1].

PFFD is characterized by a shortened and deformed femur near the hip. It becomes apparent in early childhood as a child starts crawling and transitions to attempting ambulation. PFFD can be diagnosed in utero, but its exact etiology remains largely idiopathic. Several maternal and environmental factors have been proposed to be associated with PFFD. These include maternal diabetes with poor glycemic control, exposure to certain medications such as thalidomide, viral infections during early gestation, and ionizing radiation [4, 5]. To date, no single factor has demonstrated consistent causal evidence, and most cases are considered sporadic.

The implications of PFFD extend beyond the patient’s skeletal deformity and functional restrictions to an increased burden of care and the costs of hospitalizations on caregivers and healthcare systems [6]. As such, early diagnosis and in turn early interventions are deemed necessary to address functional and structural limitations. When nonoperative approaches and prosthetic devices fail to maintain a decent quality of life, especially in severe cases of PFFD, several surgical options are available to restore limb length and correct deformities to enhance mobility and functionality but at an additional risk of complications [2]. The latter options include external fixators, internal motorized magnetic lengthening devices, rotationplasty, and SUPERhip, among others [7–11].

Although PFFD is rare, its clinical relevance extends beyond incidence due to the complexity of diagnosis, classification, and management. This case is educationally valuable because it highlights bilateral asymmetric disease with differing Aitken classifications, a scenario that challenges standard treatment algorithms. It also underscores the importance of integrating imaging modalities, classification systems, and socioeconomic considerations into individualized care planning. Such cases provide critical insights into real‐world decision‐making, particularly in resource‐limited settings. Both oral and written informed consent was obtained regarding the case study and future publications.

## 2. Case Presentation

A 2‐year‐old girl born via normal vaginal delivery at term and known to be previously healthy presented to our care for abnormal crawling, standing, and walking postures noticed by her parents since she became mobile and physically active. Prenatal and perinatal history was nonsignificant for any complications. She is the first baby to nonconsanguineous parents who deny a family history of congenital skeletal anomalies in the extended family. During the early months following birth, the patient had normal functional developmental progress until the age of 11 months when she was transitioning to the crawling phase and then to the standing/attempting‐to‐ambulate phase.

On presentation to our clinic, the patient’s anthropometric data were a weight of 18.5 kg, height of 94 cm, and body mass index of 20.9 kg/m^2^, with an apparent abnormal standing and ambulatory stance (Figure 1), in addition to unusual crawling on her hands and tiptoes with the knees not touching the floor. She was a joyful girl and not in pain. On physical examination, the patient had shortening of both thighs, which were bulky. She had an apparent limb‐length discrepancy (LLD) of 2.5 cm as measured clinically from the ASIS to the medial malleolus, with the right being shorter than the left (Figure 2). The hips were flexible but were noticed to be in flexion, abduction, and external rotation, and Thomas test was positive. There was a noticeable bilateral flexion contracture of both knees. She had a lax anterior cruciate ligament as well on the anterior drawer test. No dysmorphic features were otherwise appreciated.

**FIGURE 1:**
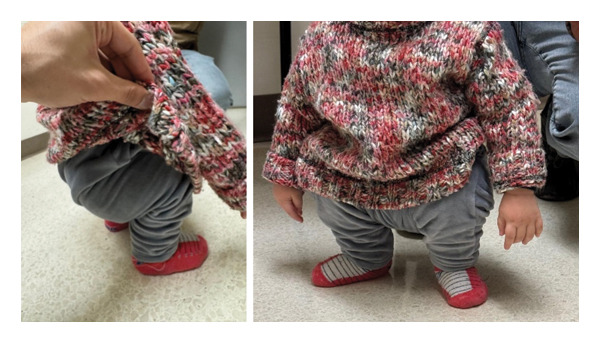
Sideway and forward views of an apparent abnormal standing stance.

**FIGURE 2:**
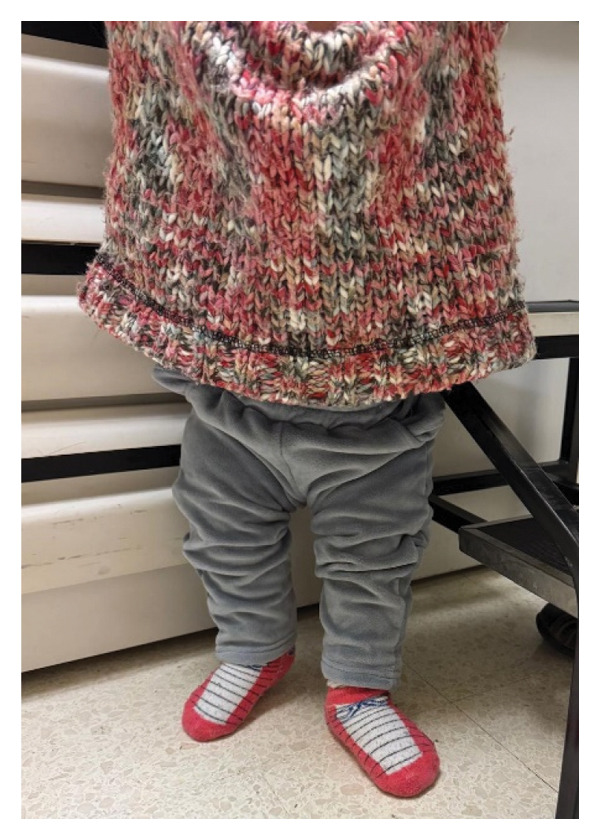
Apparent discrepancy in limb length with the right leg shorter than the left in a regular standing stance.

Upon investigation, an X‐ray of the lower extremities showed focal deficiency of the proximal femurs bilaterally in addition to shortening of the femurs as compared to the tibia length, consistent with PFFD (Figure 3). Further imaging with MRI showed bilateral severely dysplastic acetabula with dislocation of bilateral femurs and formation of pseudoacetabula (Figure 4). The right femur is consistent with Aitken Class C as the femoral head is absent and the proximal femur is tapered with no osseous connection between the shaft and proximal ossicle, and no articular relation with the acetabulum. The left femur is consistent with Aitken Class B as the femoral head is present and seen as a cartilaginous ossicle that is in contact with the acetabulum (Figure 5), but the proximal femoral bony tuft is tapered and dislocated with no osseous connection between the head and the shaft.

FIGURE 3X‐ray of the pelvis (a) showing asymmetrically shortened femurs that are dislocated with focal deficiency of the proximal femurs bilaterally and formation of pseudoacetabula (left); (b) frog‐leg X‐ray showing shortening of the femurs as compared to the tibia length consistent with PFFD.

**Figure:**
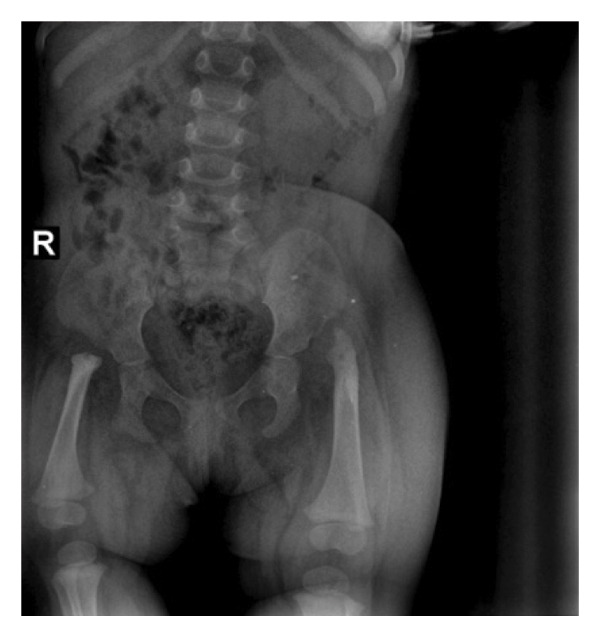
(a)

**Figure:**
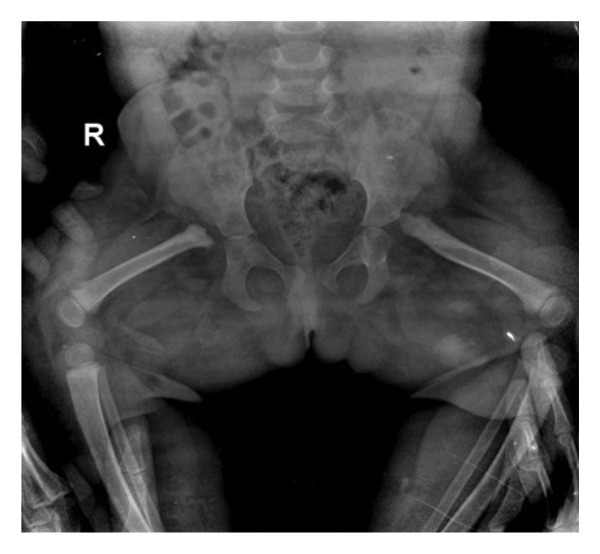
(b)

**FIGURE 4:**
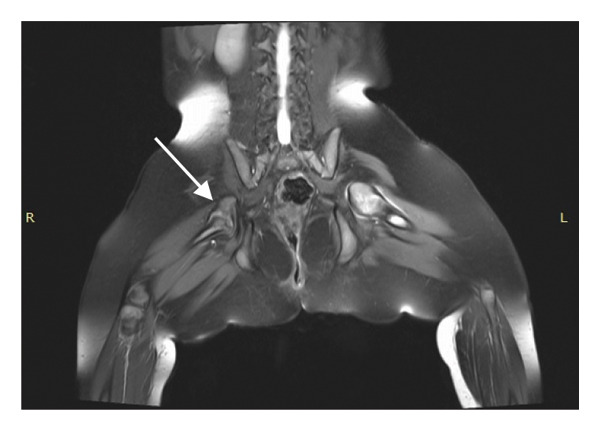
Coronal T2‐weighted MRI of the pelvis showing bilateral severely dysplastic acetabula with dislocation of bilateral femurs and formation of pseudoacetabula. The right femur is consistent with Aitken Class C, with an absent femoral head and a tapered proximal femur (white arrow).

**FIGURE 5:**
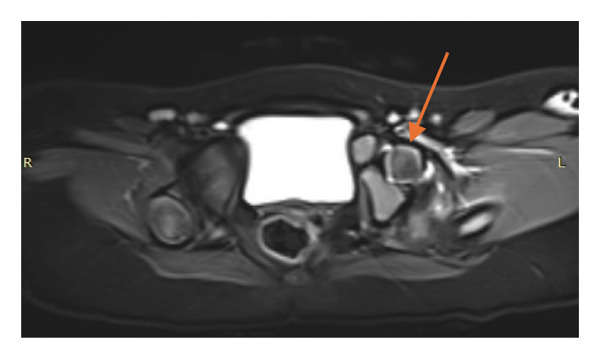
Axial T2‐weighted MRI of the pelvis showing the left cartilaginous femoral head present (orange arrow) and in contact with the acetabulum, but the proximal femoral bony tuft is tapered and dislocated with no osseous connection between the head and the shaft; consistent with Aitken Class B.

In the context of an ambulatory young preschool girl, under 3 years of age, without pain and with bilateral PFFD of 2–3 cm LLD and mild functional impairment, and given the complexity of the surgical options available, their complications, and the need for long‐term close follow‐up, and the financial constraints on the family, a discussion with the family was thoroughly performed on the nonoperative and the operative options available and their implications on the girl’s life course. The parents elected to proceed with nonoperative management, and they were instructed to perform lower extremities range of motion and exercises to maintain mobile joints and avoid contractures. Additionally, the parents were educated on the necessity of clinic visits every 6 months for clinical and radiographic assessment, the need for custom‐made prosthetics to equalize limb length and aid in ambulation, and the possibility of a future need for limb‐lengthening and joint stabilization/arthrodesis procedures when necessary (functional decline, progression of contracture, and significant limb‐lengthening discrepancy [> 3 cm]).

## 3. Discussion

This case report discloses a case of bilateral PFFD of different Aitken classifications, Class C on the right hip and Class B on the left. The associated femoral dislocation and formation of pseudoacetabula bilaterally with the accompanying functional restrictions warrant a multidisciplinary approach and thorough review and investigation of different treatment options available.

PFFD is a rare anomaly with a very low incidence [12]. It is a congenital defect in the primary ossification center, leading to hypoplasia or absence of the proximal femur. The spectrum can range from a shortened femur to an absent hip; however, a presentation as a short, bulky thigh flexed, abducted, and externally rotated secondary to an underlying shortened femur with proximal deficiency and varus deformity is pathognomonic [13]. It is sporadic in etiology, with bilateral involvement in 15% and can be associated with fibular hemimelia, anterior cruciate ligament deficiency, coxa vara, and contractures [1, 14].

Diagnosis of PFFD relies on a stepwise approach that integrates clinical evaluation with progressive imaging modalities to define anatomy, classify severity, and help guide management. When PFFD is clinically suspected due to abnormal crawling, standing, or gait with apparent LLD, or limited hip motion, imaging follows to identify the underlying cause. Initial imaging begins with plain radiographs of the pelvis and lower limbs that help identify shortening, acetabular morphology, and overall alignment [15]. Teleradiographs are valuable as they can quantify LLD, assessing coronal and sagittal plane deformities. However, in very young children, the lack of ossification in the femoral head and neck may limit the accuracy of radiographic assessment. Ultrasound is particularly useful during early infancy before ossification centers appear to evaluate the integrity of the cartilaginous femoral head and acetabulum. MRI remains the gold standard for accurate classification, preoperative assessment, and treatment planning. It is more accurate than radiographic evaluation in classifying PFFD especially in the early years prior to ossification of the cartilaginous component, provides detailed visualization of nonossified structures, including the cartilaginous femoral head, acetabular cartilage, and labral configuration, and determines the continuity—or lack thereof—between the femoral head and shaft [14, 16].

Several classifications have been proposed in the literature; Aitken’s classification is the most commonly used. Class A demonstrates a shortened femur with the femoral head attached to the shaft by a femoral neck in coxa vara with subtrochanteric pseudoarthrosis and a well‐formed acetabulum. In Class B, there is a femoral head but without a connection to the shaft that is shortened, with a proximal bulbous bony tuft, whereas in Class C, the femoral head is absent or very small, the femoral shaft has a tapered end, and the acetabulum is severely dysplastic. In Class D, there is an absence of both the femoral head and the acetabulum [17].

Paley and Nossov proposed the CFD classification to unify older systems (including Aitken and Amstutz) based on the presence of the femoral head, continuity between the head and shaft, and acetabular morphology. PFFD represents one end of the broader spectrum of CFD and corresponds primarily to the more severe CFD types, specifically Paley Type 2 and Type 3, where the femoral head is either disconnected from the shaft or completely absent, often accompanied by acetabular dysplasia and soft‐tissue contractures; in contrast, milder CFD types (Paley Type 1) retain partial continuity between the femoral head and shaft [4]. This framework helps guide treatment selection, with reconstructive options feasible for milder types and rotationplasty or prosthetic approaches often reserved for more severe forms such as PFFD.

Treatment of PFFD is tailored to disease classification and implications on quality of life and focuses on overcoming debilitating structural deformities by emphasizing limb length, gait restoration, joint stability, overall function, and independence. Conservative care is most appropriate for mild or asymptomatic cases, particularly when the hips remain flexible and pain‐free and when socioeconomic or anatomic factors limit surgical feasibility. In contrast, surgical reconstruction or lengthening is generally reserved for patients with greater functional impairment, rigid contractures, or severe bony deficiency as seen in higher Aitken or Paley CFD types. Early conservative therapy may delay or even reduce the extent of surgical intervention required later, underscoring its value within a multidisciplinary, staged management framework [18].

The primary goals of conservative management are to preserve joint motion, prevent contractures, maintain functional gait balance, and prepare the limb for future prosthetic fitting if needed. Early physiotherapy plays a central role, emphasizing passive and active range‐of‐motion exercises to prevent hip and knee stiffness and maintain muscular strength. Stretching protocols and orthotic support—including hip–knee–ankle–foot orthoses or shoe lifts—help compensate for LLD and enhance postural alignment during ambulation. Regular follow‐up is essential for monitoring growth and functional adaptation. Clinical and radiographic reassessments are typically performed every 6–12 months, with closer surveillance during periods of rapid growth. Reevaluation focuses on LLD progression, joint mobility, and development of compensatory deformities. Indications for transitioning from conservative to surgical management include significant functional limitation, persistent instability, progressive deformity, or LLD exceeding 2–3 cm despite orthotic correction [19].

The basis of bone lengthening in patients with LLD revolves around the concept of distraction osteogenesis while applying external or internal devices that maintain alignment of the bone fragments following osteotomy, hence allowing lengthening and callus formation and consolidation at the bone gap [20]. Following 7 days of latency period, lengthening at a rate of 1 mm/day is enough to achieve the desired length while keeping adequate room for bone formation and healing. The fixation device needs to be maintained until evident bone consolidation [10, 11, 20].

Lengthening with external fixation is performed by an Ilizarov apparatus or with a monolateral external fixator. The Ilizarov circular fixator is versatile, affordable, and particularly useful in young children for correcting angular deformities but is associated with a high incidence of pin site infections [21]. Complications are high, reaching up to 69%, and include loss of knee flexion and need for quadricepsplasty, hip subluxation, tibia fracture, and osteomyelitis [10, 22, 23]. A monolateral fixator is a simpler device with fewer complications and limited rotational control. In comparison to external fixation, motorized intramedullary nails show a superior range of motion and lower overall complication rates but similar distraction and healing indices [7, 24].

Several internal fixation devices were introduced. PRECICE nails are magnetically adjustable devices used in older children with wider medullary canals [21]. The use of PRECICE nail accompanied by joint reconstruction/stabilization procedures resulted in effective limb lengthening without mechanical failures, with shorter and favorable healing indices of both femurs and tibias, albeit at different rates [11]. It is accurate and minimally invasive but limited by bone size and age; complications include joint subluxation, delayed healing requiring bone grafting, soft tissue contracture, overlengthening, rod settling/breakage, and segmental fractures [7, 11, 24, 25]. Moreover, a study assessing the efficacy of prophylactic nailing following lengthening with an external fixator and frame removal found a lower incidence of femur fracture (18% vs. 34%) at an acceptable rate of nail complications [26, 27].

Brown and Paley–Brown rotationplasty are surgical alternatives indicated in cases of Paley Types 2 and 3. The procedure involves rotating the femur and attaching it to the tibia to create a functional “new knee” and to the pelvis to secure the new hip joint and prevent epiphyseal growth [8, 28]. Rotationplasty is also indicated as a last resort of reconstruction in cases of multiple failed lengthening and corrective procedures [29]. Postoperatively, and until healing, patients require cast application or bed rest followed by nonweight bearing for 12 weeks before which prosthetic devices can be fitted and gait training started. On long‐term follow‐up, Van Nes rotationplasty resulted in a good quality of life and a high functional level but reduced symmetry in stance/posture, and slower gait and walking speed, and a high complication rate reaching 60% including wound dehiscence and flap necrosis, sciatic nerve palsy, delayed union, and corrective osteotomies [30]. SUPERhip is a procedure performed below 5 years of age as a preparatory surgery prior to lengthening. It was developed in 1997 by Paley et al. to achieve a stable hip joint and involves soft‐tissue lengthening to correct flexion and abduction contracture, proximal femur osteotomy to correct flexion, varus, and torsional deformities, and, when necessary, acetabular osteotomy to correct dysplasia [9, 31]. Complications amount to 15% and involve persistent varus deformity and delayed ossification.

It is important for clinicians and surgeons to take care of multiple factors. During femoral lengthening, it is preferable to perform the osteotomy in the metaphyseal region, namely the subtrochanteric and supracondylar regions, for their wider bone structure and favorable bone regeneration [32]. Previous studies reported growth inhibition of the physes of the distal femur and proximal tibia of the affected limb, which, after lengthening, exhibit greater net growth stimulation and hence greater than expected lengthening [10, 22, 33]. In case of overlengthening where LLD is anticipated, epiphysiodesis can be effective at a later stage [22]. Physical therapy is of paramount importance during distraction osteogenesis; a mobile knee of at least 45° flexion and full extension is necessary. In case of decreased knee flexion or tibiofemoral subluxation, the lengthening rate may need to be adjusted or stopped [10]. Despite a temporary decrease in hip and knee range of motion during the lengthening phase, it is expected to return to the preoperative state at 2–3 years. Severe cases of PFFD may be beyond surgical correction and in turn warrant approaches such as selective amputation for prosthetic fitting facilitation [34]. It is not uncommon, however, for the guardians to choose conservative nonsurgical approaches to treat their child. Hence, it is important to explain that there is room for the latter approach in select cases of PFFD using custom‐made prosthetics associated with physical therapy and muscle stretching and strengthening protocols, serial follow‐up, and clinic visits [35].

Management in PFFD should follow a structured decision‐making framework that includes anatomic assessment (femoral head presence, hip stability, and acetabular development), functional status (mobility, pain, and joint range of motion), predicted LLD at maturity, age and growth potential, family preferences, and socioeconomic factors. Table 1 provides a mini‐review of treatment options available to highlight indications, advantages, and complications (Table 1). In our patient, several factors favored conservative management including mild functional impairment and the absence of pain at a young age (< 3 years), limiting surgical feasibility in addition to bilateral involvement complicating reconstructive symmetry and financial constraints affecting access to staged surgical care. Therefore, nonoperative management with physiotherapy, orthotic support, and surveillance was deemed appropriate, with the option of delayed surgical intervention as the child grows and functional demands increase.

**TABLE 1:** A review of management options available for PFFD.

Modality	Indications	Advantages	Limitations/complications
Conservative (PT + orthotics)	Mild deformity, good mobility	Non‐invasive, preserves function	Does not correct deformity
External fixation (Ilizarov)	Moderate–severe LLD	Versatile, deformity correction	High complication rate (infection, stiffness)
Intramedullary lengthening (PRECICE)	Older children, adequate canal	Better ROM, fewer infections	Age/bone size limitations
SUPERhip procedure	Young children with instability	Improves hip stability	Requires expertise
Rotationplasty	Severe deformity (Paley 2‐3)	Good long‐term function	Cosmetic concerns, major surgery
Amputation + prosthesis	Nonreconstructable cases	Reliable function	Psychological impact

While most reports describe unilateral PFFD, bilateral cases—particularly asymmetric ones—are sparsely represented in the literature. Previous studies have emphasized that treatment strategies differ significantly depending on femoral head presence, hip stability, and acetabular development, reinforcing the utility of MRI‐based classification in early childhood [16]. Furthermore, recent advances in limb‐lengthening technologies, including magnetically controlled intramedullary nails, have shifted management paradigms toward staged reconstruction in selected patients, although their applicability remains limited in younger children with severe deformities. This case contributes to the literature by demonstrating how discordant bilateral anatomy necessitates hybrid decision‐making rather than uniform intervention. Individualized treatment of PFFD remains a key factor when selecting the appropriate surgical option to compensate for the anatomical and biomechanical deficiencies [34]. A variety of options exist nowadays to address such deformities; however, a multidisciplinary approach that combines medical expertise and patient‐centered care is necessary to discuss these options with the family and to tailor the treatment in an individualized fashion based on the patient’s disease characteristics, deformities, functional restrictions, and socioeconomic factors to optimize long‐term outcomes and quality of life. Further research is needed to explore the safest treatment approach for patients in terms of mobility, limb length restoration, and quality of life at a low complication rate.

### 3.1. Teaching Points

• Bilateral PFFD is a rare entity that is associated with a myriad of biologic, functional, and social difficulties.
• PFFD is part of the *CFD spectrum*, requiring classification‐driven management.
• *MRI is the gold standard* in early childhood for accurate classification due to unossified structures.
• Bilateral PFFD may present *asymmetrically*, requiring independent evaluation of each limb.
• Management decisions must integrate functional status, classification severity (Aitken/Paley) and socioeconomic context.
• Early conservative management can *preserve joint mobility and delay invasive procedures*.
• Multidisciplinary care is essential for optimizing long‐term functional outcomes.

## 4. Conclusion

Bilateral asymmetric PFFD represents a complex manifestation within the CFD spectrum that challenges conventional diagnostic and therapeutic pathways. Despite its rarity, PFFD emphasizes the importance of early, structured imaging—particularly MRI—for accurate classification, which directly informs management decisions. It also highlights that treatment should not be uniform even within the same patient, as each limb may require independent evaluation. A staged, multidisciplinary, and individualized approach—balancing anatomical severity, functional status, and socioeconomic factors—is essential. Treatment continues to be a topic of controversy that requires a multidisciplinary approach to assess treatment paradigms and societal factors. Early conservative management remains a valid strategy in selected patients, allowing preservation of function while maintaining flexibility for future reconstructive interventions including reconstruction and external fixation or intramedullary nailing or the SUPERhip procedure, with rotationplasty and amputation reserved for severe, nonreconstructable cases.

## Author Contributions

Ahmad Hammad and Rachid Haidar contributed to conceptualization of the study. Ahmad Hammad, Haya El Merkabaoui, Mohammad Kassem, and Claudia El Haddad contributed to manuscript writing. Hicham Moukaddam and Rachid Haidar contributed to critical analysis.

## Funding

No funding was received for this manuscript.

## Disclosure

All authors reviewed the manuscript.

## Consent

The authors certify that they have obtained all appropriate patient consent forms. In the form, the patient has given the consent for his/her images and other clinical information to be reported in the journal. The patient understands that his/her names and initials will not be published and due efforts will be made to conceal their identity, but anonymity cannot be guaranteed. Written informed consent was obtained from the patient for publication and any accompanying images. A copy of the written consent is available for review by the editor‐in‐chief of this journal on request. Both oral and written informed consent was obtained regarding the case study and future publications.

## Conflicts of Interest

The authors declare no conflicts of interest.

## Data Availability

The data that support the findings of this study are available on request from the corresponding author. The data are not publicly available due to privacy or ethical restrictions.
